# Characterizing the effect of short wavelengths on the floral flavonoid metabolome of medicinal cannabis using a comparative computational metabolomics workflow

**DOI:** 10.1007/s11306-026-02500-x

**Published:** 2026-07-01

**Authors:** Willy Contreras-Avilés, Laura Rosina Torres-Ortega, Ep Heuvelink, Leo F. M. Marcelis, Justin J. J. van der Hooft, Iris F. Kappers

**Affiliations:** 1 Horticulture and Product Physiology, Plant Sciences Group, Wageningen University, P.O. Box 16, Wageningen, 6700 AA The Netherlands; 2 Plant Physiology, Plant Sciences Group, Wageningen University, P.O. Box 16, Wageningen, 6700 AA The Netherlands; 3 Bioinformatics Group, Wageningen University, Wageningen, P.O. Box 16, Wageningen, 6700 AA The Netherlands; 4 Department of Biochemistry, University of Johannesburg, Johannesburg, 2006 South Africa

**Keywords:** UVB, Flavonoids, Inflorescence, Glycosylation, Metabolomics, Medicinal cannabis

## Abstract

**Background:**

Controlled-environment cultivation of medicinal cannabis (*Cannabis sativa* L.) typically optimizes light conditions to enhance the biosynthesis of pharmaceutically important metabolites like cannabinoids. Such experimental strategies may also influence other specialized metabolites like terpenoids, flavonoids, alkaloids, among others. Previous untargeted metabolomics studies testing short-wavelength conditions like UV and blue light have shown that terpenoids and prenylated flavonoids in cannabis leaves respond differentially. However, since metabolomic studies in cannabis have so far mostly focused on floral cannabinoids, a comprehensive untargeted study into cannabis’ floral metabolome response to short wavelengths is currently lacking.

**Objectives:**

Our study investigates the impact of short-wavelength usage on cannabis specialized metabolism, and in particular the influence of UVB, UVA, and blue light on the cannabis floral flavonoid metabolome and associated glycosylation moieties.

**Methods:**

Cannabis plants were grown under a white background light and exposed to supplemental UVB, UVA, or blue light during the generative phase of the cultivation cycle. Treatments were compared to a reference white background light without UV or blue light. Metabolites from floral tissue were extracted and analysed via ultra-performance liquid chromatography-tandem mass spectrometry. A comparative metabolomics workflow was designed and used to characterize the floral flavonoid metabolome and associated glycosylation moieties.

**Results:**

Our results demonstrate how short wavelengths differentially affect the metabolism of natural product compound classes including polyketides and phenylpropanoids/shikimates. Blue light induced flavonoids similarly to how UVB did, while both UVA and blue light specifically induced flavanones accumulation. UVB showed the strongest regulatory effect on flavonoids production and glycosylation patterns.

**Conclusions:**

UVB reshapes the cannabis floral flavonoid metabolome by selectively stimulating the accumulation and structural modification of flavonoids. Therefore, UVB represents a potential horticultural strategy to enhance flavonoid-related aspects of medicinal cannabis inflorescence phytochemical quality, without affecting cannabinoid levels.

**Supplementary Information:**

The online version contains supplementary material available at https://doi.org/10.1007/s11306-026-02500-x.

## Introduction

*Cannabis sativa* L., is a medicinal plant producing health-affecting specialized metabolites including cannabinoids, terpenoids, flavonoids, as well as unique flavo-alkaloids and prenylated flavones (cannflavins) (Jin et al., 2020; Muller & De Villiers, 2025; Rea et al., 2019). Cannabinoids and terpenoids predominantly accumulate in cannabis inflorescences, whereas flavonoids, flavo-alkaloids, and cannflavins also can be found in leaves (Jin et al., 2020). The accumulation of cannabis specialized metabolites can be influenced in controlled-environment agricultural systems where light and other environmental factors are largely under control (Contreras-Avilés et al., 2024; Desaulniers Brousseau et al., 2021). Light is an essential environmental factor consisting of photons that once perceived by cryptochromes (CRYs) and phytochromes (PHYs) trigger a signalling cascade driving photomorphogenesis and specialized metabolism (McCree, 1981; Opálková et al., 2018). Shorter wavelengths including UVB (280–315 nm) and UVA (315–400 nm) influence growth, morphology, and plant specialized metabolism via UV RESISTANCE LOCUS 8 (UVR8) and CRYs photoreceptors, respectively (for details see 1.1 Introduction - Supplementary Information) (Contreras-Avilés et al., 2024; Rai et al., 2021; Sun et al., 2024, 2025). Specialized metabolites are of great anthropocentric interest due to their nutritional and medicinal properties (for details see Supplementary Information − 1.2 Introduction -) (Contreras-Avilés et al., 2024). The flavonoids specialized metabolite class (for details see Supplementary Information 1.3 Introduction -) is widely induced under UV radiation (Rai et al., 2021), and has been described to have multifunctional roles in plants including photoprotection, antioxidant activity, and signalling (Agati & Tattini, 2010; Mao et al., 2025; Patil et al., 2024). Flavonoids originate from the phenylpropanoid pathway, where phenylalanine-derived *p*-coumaroyl-CoA is combined with malonyl-CoA units via chalcone synthase to form the core flavonoid skeleton. Subsequent enzymatic modifications diversify this core structure into the major flavonoid subclasses flavanones, flavanols, anthocyanins, flavonols, and (iso)flavones, which are often found as hydrophilic glycosides or lipophilic methyl/prenylated/geranylated molecules (Shomali et al., 2022). Among flavonoids, flavonols function as specialized metabolites that mitigate short-wavelengths-induced stress through reactive oxygen species (ROS) scavenging, metal chelation, inhibiting ROS-generating enzymes, and as cofactors of antioxidant enzymes (Shomali et al., 2022). UVB has been shown to induce flavonols as kaempferol in *Coriandrum sativum* (Fraser et al., 2017) and quercetin in *Ginkgo biloba* (Zhao et al., 2020) as well as glycosylated derivatives (for details see Supplementary Information − 1.4 Introduction) in *Asparagus officinalis* L. (Eichholz et al., 2012). Similarly, increased solar radiation enhanced quercetin-3-O-rutinoside and luteolin-7-O-glucoside in *Ligustrum vulgare* (Tattini et al., 2004), while combined UV (UVB + UVA) and blue light promoted flavonol disaccharides in *Cucumis sativus* (Palma et al., 2021).

Short wavelengths (< 500 nm) promote ROS and regulate accumulation of glycosylated flavonoids via UVR8 signalling (Markovitsi et al., 2013; Nishigori et al., 2023; Rai et al., 2021). However, responses are species and structure-dependent, for example, UV increased kaempferol glycosides but decreased quercetin glycosides in *Brassica oleracea* (Moreira-Rodríguez et al., 2017; Rechner et al., 2016), while shifting mono- and di-glycosylated flavonoids in *Glycyrrhiza uralensis* (Zhang et al., 2018). UVB enhanced either kaempferol glycosides or quercetin glycosides depending on species (*Brassica rapa*,* B. oleracea*,* B. carinata*) (Neugart & Bumke-Vogt, 2021). UVA increased kaempferol glycosides in *Glycine max* (Lim et al., 2021), and flavone glycosides in *Scutellaria baicalensis* (Miao et al., 2022), while blue light promoted flavone glycosides in cereals and anthocyanins in *Malus domestica* (Kokalj et al., 2019; Muthusamy et al., 2020). Overall, glycosylated flavonoid responses appear structure-dependent (Neugart & Schreiner, 2018), and it remains unclear whether UVA and blue light elicit distinct effects despite sharing CRYs signalling pathways (Rai et al., 2019).

Flavonoids represent approximately 80% of the phenolic compounds detected in cannabis inflorescences, and these compounds are associated to anti-inflammatory, antioxidant, neuroprotective and anticancer activities (Bautista et al., 2021; Izzo et al., 2020). Therefore, cannabis medicinal quality is not determined solely by cannabinoids and terpenoids, but flavonoids as well (Jin et al., 2020). Rather few studies have investigated the effects of UV and/or blue light on flavonoid accumulation in cannabis, and those available lack detailed analysis of flavonoid profiles and glycosylation patterns (Kotiranta et al., 2024; Marti et al., 2014). Although flavonoids are abundant in leaves, their occurrence in inflorescences appears to be cultivar-dependent or associated with oxidative stress (Desaulniers Brousseau et al., 2021), and their role in floral light responses remains poorly understood. Addressing this knowledge gap requires comprehensive metabolites profiling approaches. Computational metabolomics (“cannabinomics”) has been widely used to characterize cannabis chemical diversity and support chemovar classification, breeding, and bioactivity studies (Aliferis & Bernard-Perron, 2020; Cerrato et al., 2023; Liu et al., 2025; Myoli et al., 2024; Tang et al., 2023; Vásquez-Ocmín et al., 2021). However, these studies have primarily focus on cannabinoids and terpenoids, with flavonoids receiving little attention. Untargeted UPLC–MS/MS can yield thousands of chromatographic features for comparative analyses, but translating these signals into confident chemical identities remains a key bottleneck, particularly for flavonoids and their glycosylated forms, often occurring as positional isomers with highly similar MS/MS patterns and limited spectral library coverage (Bittremieux et al., 2022; Li et al., 2024). Therefore, using cannabis inflorescences from a prior short-wavelength exposure experiment, we created and applied an integrated computational workflow (mzmine–MN–MS2LDA–SIRIUS–MS2Query–DreaMS) to characterize the floral flavonoid metabolome, linking differential abundance patterns to molecular families (MN), substructures and glycosylation moieties (MS2LDA), chemical class assignments (SIRIUS/CANOPUS), and structural analogues via machine and deep learning similarity (MS2Query; DreaMS) (for details see Supplementary Information − 1.5 Introduction) (Bushuiev et al., 2025; De Jonge et al., 2023; Dührkop et al., 2019; Torres Ortega et al., 2025). We hypothesize that short wavelengths differentially promote flavonoid glycosylation as an antioxidant strategy under oxidative stress in medicinal cannabis inflorescences. Our results specifically show that UVB induces flavonol accumulation and enhances glycosylation, while UVA and blue light elicit subclass-specific responses.

## Materials and methods

We used *Cannabis sativa* L. (var. King harmony; Chemotype II, balanced CBD:∆^9^-THC) (Perfect plants, Honselersdijk, the Netherlands) inflorescences collected in a previous study (Contreras-Avilés et al., submitted). In short: plants were grown for 12 days under a white (10% Blue, 10% Green, 80% Red) background light and a 18 h photoperiod (400 µmol m^− 2^ s^− 1^) to stimulate vegetative growth, after which the flowering phase was initiated by switching to a 12 h photoperiod (600 µmol m^− 2^ s^− 1^). Light treatments started 14 days after the short-day phase was initiated by exposing plants to supplemental UVB (2 µmol m^− 2^ s^− 1^; 3 h d^− 1^), UVA (88 µmol m^− 2^ s^− 1^; 12 h d^− 1^), or blue (84 µmol m^− 2^ s^− 1^; 12 h d^− 1^) light; all treatments were applied at the beginning of the photoperiod. UVA and blue intensity and photoperiod were matched to ensure comparability, while UVB parameters were selected to minimize harm while stimulating specialized metabolism. All treatments were compared to a reference without supplemental light. To evaluate the coloration of glandular trichomes, these anatomical structures were imaged (see Supplementary Information - Methods 2.1).

### Ultra-high-performance liquid chromatography mass spectrometry (UHPLC-MS/MS) based metabolomic analysis

At maturity (eight weeks after the onset of the generative phase) inflorescence samples were taken from within 5 cm of the apical section of the top inflorescences and flash frozen in liquid nitrogen. One inflorescence sample per replicate plant was collected. Each experimental unit was composed by nine plants, and from each light treatment six independent replicates plants were randomly taken for metabolic extraction. Frozen samples were freeze-dried (Alpha 1–4 LSCbasic freeze-dryer, Martin Christ, Osterode am Harz, Germany) for 36 h and ground into a fine powder (MM 200 mixer mill, Retsch, Dale i Sunnfjord, Germany). Approximately 20 mg of powdered inflorescence material was extracted with 500 µL of 95% ethanol and sonicated (Branson 2800 ultrasonic bath) for 10 min in an ice-bath. Pellets were re-extracted with 200 µL methanol (20% v/v) with formic acid (0.1% v/v). Combined supernatants were dried (SpeedVac SPD2030-230, Savant, Thermo Scientific, USA) and reconstituted in 80% MeOH (0.01% formic acid); sonicated for 30 s and centrifuged at 21,000×g at 4 °C for 10 min prior to injection (see Supplementary Information - Methods 2.2).

### Data preprocessing and metabolite feature finding

The acquired raw datasets (raw, ESI positive and negative ionization mode centroid data) were converted to.mzML using ProteoWizard MSConvert (3.0) and pre-processed using mzmine 4.7.8. (see Supplementary Information - Methods 2.3). After filtering, 5,700 (positive ionization mode) and 8,664 (negative ionization mode) features were retained.

### Metabolite and substructure in silico annotation

**Fig. 1:**
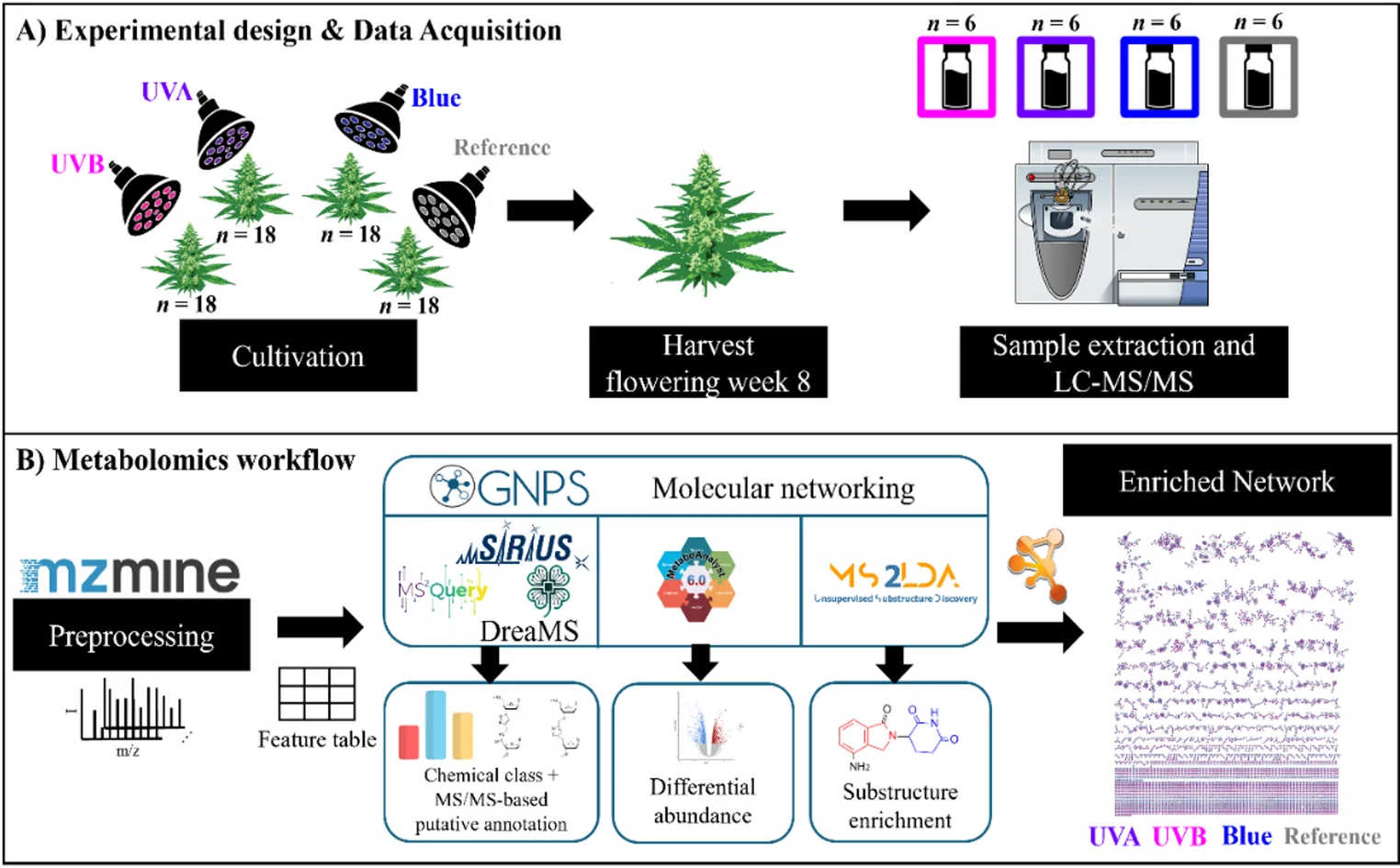
Comparative computational metabolomic workflow. Building on a previous short-wavelength experiment, **A** cannabis plants were exposed to UVB, UVA, and blue light treatments, and inflorescence samples were taken eight weeks after the onset of flowering, followed by UPLC-MS/MS analysis. **B** mzmine was used to preprocess and produce a feature table which was also complementarily used for GNPS FBMN and MS/MS-based putative annotation (SIRIUS/CANOPUS. MS2Query, DreaMS). Differential abundance analysis (MetaboAnalyst) and substructure discovery (MS2LDA) were also performed. Altogether, the results were integrated into an enriched network visualized in Cytoscape prioritizing flavonoid metabolites features

The output from mzmine was used for in silico annotation using SIRIUS (Dührkop et al., 2019) for molecular formulas and ZODIAC (8.5.6) (Ludwig et al., 2020) to refine assignments. Chemical class annotations were assigned with CANOPUS as a part of the SIRIUS framework (Dührkop et al., 2019). The settings used for SIRIUS are described in Supplementary Information - Methods 2.4. To complement these annotations, we applied two similarity-based spectral annotation approaches. First, MS2Query (De Jonge et al., 2023) was used to retrieve candidate library annotations for each query spectrum based on learned MS/MS similarity and re-ranking, providing putative identifications. Second, DreaMS (Bushuiev et al., 2025) was applied as an embedding model to perform nearest-neighbour spectra similarity, obtaining the most similar reference spectra above a cosine similarity threshold of 0.75 (via the DreaMS web interface). The output of both tools includes the chemical classes inferred by applying NPClassifier to the associated candidate structures and nearest neighbour annotations (Kim et al., 2021) (Fig. 1). Substructure-level annotation was done using MS2LDA 2.0 (Torres Ortega et al., 2025) (more details in Supplementary Information - Methods 2.4).

### Feature-based molecular networking

Feature-based molecular networking (FBMN) was performed in GNPS2 (Nothias et al., 2020) using the mzmine.mgf, the feature quantification table, and a sample metadata file as inputs. The settings for the spectral library matching are described in detail at the 2.5 – Supplementary Information section. The resulting network was exported as a GraphML file and imported into Cytoscape (v3.10.3) (Shannon et al., 2003) for visualization, annotation outputs were integrated as node attribute tables in Cytoscape using the shared feature identifier (see Supplementary Information 2.5 Methods –).

### Chemical classification and chemometric analysis

The experiment was set up and analysed as a complete randomized design, where six biological samples were considered for every treatment. Five dataset tables were generated from positive ionization data including: *global* (all features), *phenylpropanoid/shikimate*, *phenylpropanoid*, *flavonoids*, and *final annotation* (manually annotated flavones based on the Metabolomics Standards Initiative [MSI]-levels, flavonols, and anthocyanins, with duplicates and isomers removed). Datasets were pre-processed in MetaboAnalyst 6.0 (Pang et al., 2021) using left-censored data estimation, median normalization, log₁₀ transformation, and Pareto scaling. Partial least squares discriminant analysis (PLS-DA) with five-fold cross-validation, Variable Importance in Projection (VIP) scores, and hierarchical clustering heatmaps were used to visualize treatment effects. Features with VIP ≥ 1.5 were considered significant, and ≥ 2.5 as strongest drivers. Mean separation of relative abundances was performed by one-way ANOVA with Fisher’s protected LSD test (α = 0.05), and treatment effect magnitude was assessed by volcano plot analysis (more details in Supplementary Information – Methods 2.6).

## Results

A comparative metabolomic workflow was applied to characterize the cannabis floral metabolome. We first explored the mapped treatment-responsive chemical space using feature-based molecular networking and class-level annotations to identify molecules most affected by UVB, UVA, and blue light. Secondly, we focused on the phenylpropanoid/shikimate pathway to identify various chemical compound classes connected to this pathway and compared their relative abundances in response to the different short-wavelength treatments. Thirdly, we resolved flavonoid subclasses and glycosylation types (O- vs. C-glycosides) by combining structural information obtained from molecular family grouping, substructure motifs, and candidate structure annotations.

### Metabolomic profiling of cannabis floral metabolome

**Fig. 2:**
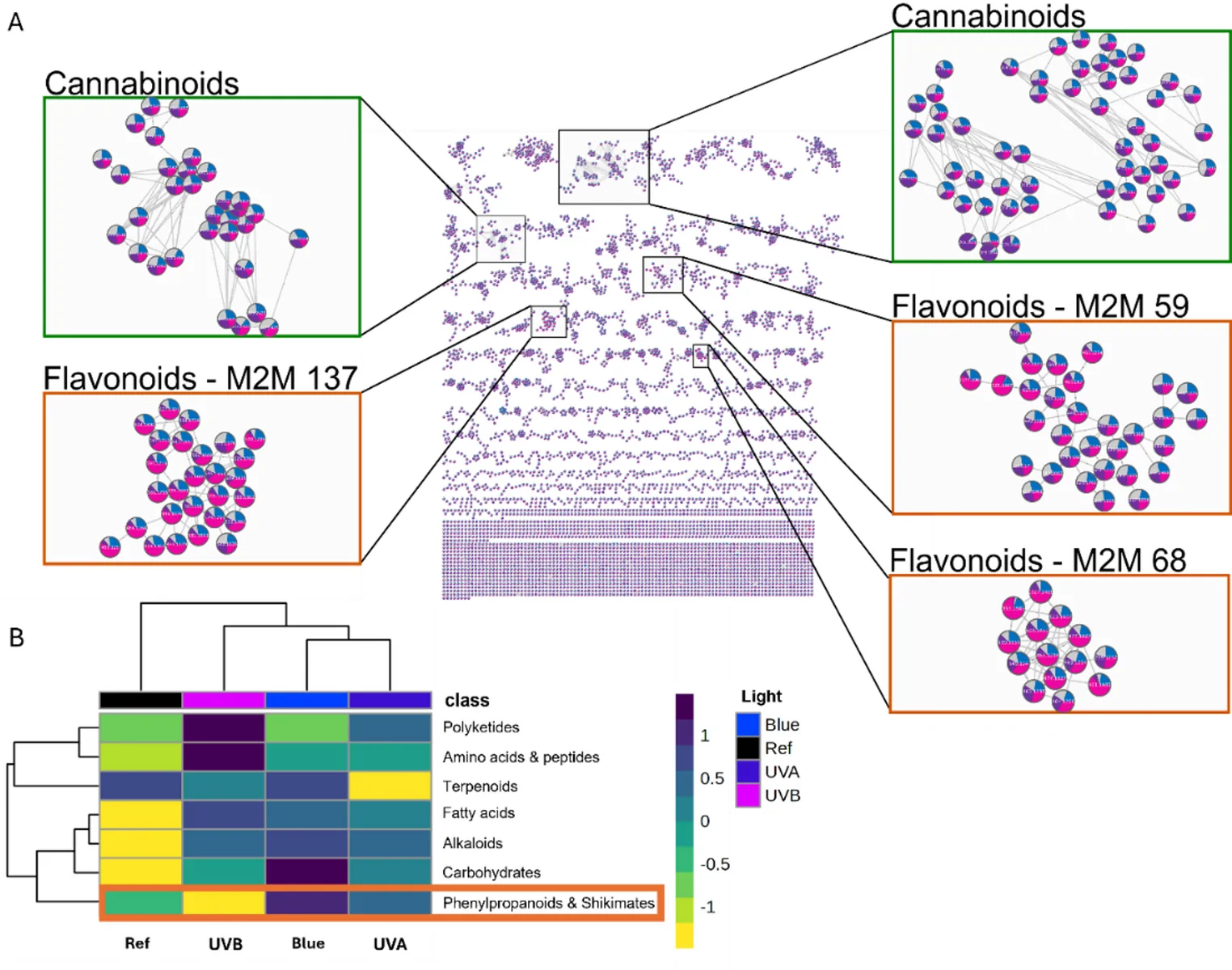
Effect of short wavelengths (UVB, UVA, blue light) on the global metabolome in *Cannabis sativa* L. inflorescences collected eight weeks after the onset of flowering. **A** Feature-based molecular network (FBMN) in positive ionisation mode: mass spectral molecular network of the flowers indicating flavonoids and cannabinoids clusters. Pie charts represent relative abundances for each feature (compound) according to the feature-based molecular network in positive ionization (FBMN-POS), where each color represents UVB (pink), UVA (purple), blue (blue), and reference (grey) short-wavelength treatments. **B** Heatmap based on the hierarchical clustering of the ‘*global*’ dataset of the floral metabolome with a VIP score ≥ 1.5; the yellow to dark blue bar corresponds to a Z-scaling ranging from − 1 to 1; the orange rectangle highlights the phenylpropanoid/shikimate subgroup

The spectra were pre-processed using mzmine to obtain the quantitative abundance of the metabolic features, and the global feature-based molecular network generated from the positive-ionisation data contained 5,700 features connected by 7,400 edges (Fig. 2A) revealed multiple molecular families covering diverse biosynthetic pathways. Based on VIP scores ≥ 1.5, short-wavelength treatments affected the cannabis metabolome in seven major chemical classes: polyketides, amino acids and peptides, terpenoids, fatty acids, alkaloids, carbohydrates, and phenylpropanoid/shikimates, according to NPClassifier annotation (Fig. 2B). Across the network, 42% of nodes had a match to entries in public structural and spectral libraries, indicating a substantial uncharacterised chemical space in cannabis flowers.

To increase putative coverage beyond library matches, we applied complementary in silico tools. SIRIUS returned 183 features (3.2%) with structures above 0.6 of confidence score, a commonly used confidence threshold for formula-to-structure assignments in datasets with limited spectral coverage (Dührkop et al., 2019). MS2Query provided analogue-level similarity (score > 0.8) for 22.35% of features. Using DreaMS (also for analogues) with a similarity cut-off of 0.75, annotations for 264 features (4.61%) were obtained. Together, these approaches expanded the interpretable cannabis inflorescence chemical space while still reflecting that a large fraction of the floral metabolome remains only partially characterised.

The negative-ion FBMN contained 8,664 nodes, but only 3.5% of these had a library match, compared to a 42% match rate in positive mode. Beyond this difference in library coverage, positive mode is particularly well-suited for characterizing specific flavonoid subclasses. For example, isomeric C-glycosylated flavones (e.g., vitexin, orientin) yield more informative fragmentation patterns when derived from [M + H]^+^ precursors (Maurer, 2010; Pereira et al., 2005). Additionally, anthocyanidin-type features are preferably detected in positive mode (Lopes-da-Silva et al., 2002). Finally, at the time of this analysis, the Mass2Motif Annotation Guidance (MAG) capability within MS2LDA was only compatible with positive ionization mode reference spectra. Therefore, the positive-ion network provides a more reliable substructure layer on which to focus our downstream annotations. The negative mode data has been deposited for future reanalysis when hopefully more reference mass spectra are available in negative ionization mode and once MAG supports negative ionization mode (see Data Availability).

Given their medicinal relevance, we first evaluated cannabinoid-related molecular families. Cannabinoid features did not differentiate across short-wavelength treatments (Fig. 2A), indicating they did not respond in this study. Cannabinoid substructures were nonetheless captured by several Mass2Motifs: in molecular family MF1, motif_102 and motif_120 associated with features annotated as cannabidiol (CBD) and cannabigerol (CBG), both containing the fragment 193.12 m/z from side-chain cleavage of the six-carbon ring (Citti et al., 2019; Ferrer, 2020) (Supplementary Fig. 1 A, B). In MF2, motif_44 and motif_9 associated with cannabidiolic (CBDA) and cannabigerolic acid (CBGA) features (Supplementary Fig. 1 C, D), where motif_44 is dominated by the 341.21 m/z fragment (water loss from the carboxylic acid) and motif_9 by 219.10 m/z (C–C bond cleavage).

Given our central hypothesis regarding short-wavelength regulation of flavonoid-like molecules, we next prioritised phenylpropanoid/shikimate-related molecular families that were both treatment-responsive (VIP ≥ 1.5) and enriched in flavonoid-like substructures. Substructure analysis with MS2LDA identified 150 Mass2Motifs across the dataset. Notably, one of the most frequent motifs (motif_87) (Supplementary Fig. 2) returned a recommended structure corresponding to four linked glycosidic moieties, with motif containing fragments related to a hexose moiety and present in 849 spectra. From MotifDB (database with manual annotations) we obtained a hit to “fragments indicative of a glycosylation” for motif_24 and motif_45 from MassBank and GNPS respectively with a score of 0.396. As this motif occurred broadly without a flavonoid core, it could imply one or possibly more sugar conjugations across multiple pathways in floral specialised metabolism.

The dominant Mass2Motifs enriched among 42 flavonoid-related nodes and particularly informative for resolving phenylpropanoid/shikimate responses include motif_59, motif_137, motif_68, and motif_105 (Supplementary Fig. 3–6). Throughout this section, MotifDB cosine matches are used as corroborating evidence alongside diagnostic fragments and MAG recommendations: scores above ~ 0.5 indicate substantial motif overlap suitable for direct annotation reuse (Rogers et al., 2019) while lower cosines reflect partial overlap of feature-probability distributions and were retained when consistent with the diagnostic fragmentation independently observed in the spectra.

Motif_59 captured the canonical flavonoid scaffold and occurred in putatively annotated aglycones such as luteolin ((MSI)-level 2) and kaempferol (MSI-level 2) (diagnostic fragment around *m/z* 287), matching MassBank motif_42 manually annotated as “fragments indicative for kaempferol” (score of 0.5875), strengthening our assignment. Motif_137 associates with glycosylated flavonoids, showing diagnostic sugar-related fragments (e.g., *m/z* 329), and was repeatedly detected in UVB-responsive nodes; the assignment rests primarily on this fragmentation pattern, with partial-overlap MotifDB hits to MassBank motif_16 (‘quercetin/glycosylated quercetin fragments’, 0.28) and motif_42 (‘kaempferol fragments’, 0.28), providing additional consistency rather than independent confirmation. Motif_68 reflected a flavonoid-like ring system with a characteristic fragment around *m/z* 301, with a partial-overlap MotifDB hit annotated as “5-Hydroxy-2,2-dimethyl-4-oxo-3,4-dihydro-2H-chromen-7-yl” (with a score of 0.194). Finally, motif_105 represented simplified aromatic-ring substructures (*m/z* 127, 135, 139), with a partial overlap match to MassBank motif_42 (‘kaempferol fragments’, score 0.23), reflecting the aromatic fragmentation shared across the flavonoid family.

Besides flavonoid-related Mass2Motifs, we have checked the motifs with the highest degree of associated number of spectra (apart from motif_87) across the entire dataset: motif_139, motif_114, motif_115, and motif_15 (Supplementary Fig. 7A-D). Motif_139 (in 1,330 spectra) was consistent with a six-membered ring containing an alkyl side chain with multiple optimised fragments, when comparing to MotifDB, the top match was annotated as “diterpenoid” (score = 0.277) from the Euphorbia Plant Mass2 MotifSet. Motif_114 was detected in 585 spectra, containing the two largest neutral losses (18.01 and 36.02 Da, with probabilities of 0.35 and 0.34, respectively). With a lack optimised fragments or loses, we propose this may represent a noisy Mass2Motif, as we are unable to associate it with a substructure. Motif_115 represented a glycosylation by MAG, which correlated with the hits against MotifDB as for motif_87 (motif_45 from GNPS and motif_24 from MassBank MotifSets respectively), indicative for glycosylation with a score of 0.574. Finally, for motif_15 (433 spectra) the three MAG recommendations contain a *p*-coumaroyl ester typically producing the diagnostic fragments 147.04 *m/z* and with the loss of CO the 119.04 *m/z* fragment; from MotifDB the hits were motif_20 and motif_37 from GNPS and MassBank (same annotation) described as “fragments for cinnamic/hydroxycinnamic acid substructures”. As *p*-coumaroyl ester is a hydroxycinnamic acid conjugated with an ester, this motif is therefore well annotated by MS2LDA. Altogether, we were able to see the effect of glycosylation in the most frequent motifs found across the dataset (Supplementary Table 1).

### Flavonoid abundances are differentially affected by UVB, UVA, and blue light

**Fig. 3:**
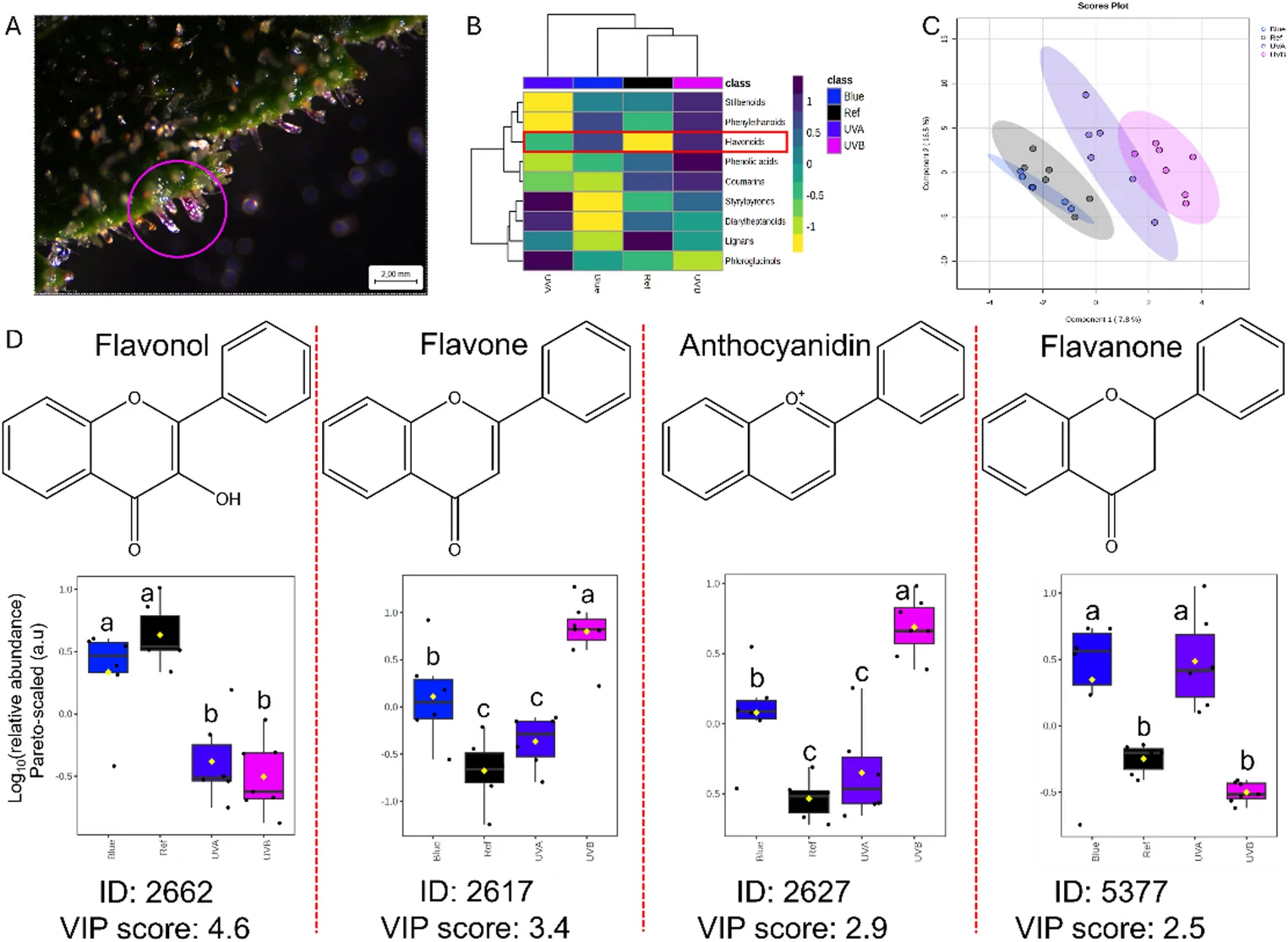
Effect of short-wavelength (UVB, UVA, blue light) on flavonoids production in *Cannabis sativa* L. inflorescences **A** Glandular trichomes on bracts in inflorescences of generative plants (eight weeks after onset of flowering) exposed to UVB. Pink circles indicate glandular trichomes with magenta coloration; **B** Partial least square - discriminant analysis (PLS-DA) of the phenylpropanoid/shikimate dataset (cross-validation R^2^ = 0.98, Q^2^ = 0.52). Ellipses represent 95% confidence region for each short-wavelength treatment; **C** Heatmap based on hierarchical clustering of the ‘*phenylpropanoid*’ dataset with a VIP score ≥ 0.5; yellow to dark blue bar corresponds to a Z-scaling ranging from − 1 to 1, orange rectangle highlights the flavonoids subclass; **D** Relative abundances of flavonoid features within the ‘*phenylpropanoid/shikimate*’ dataset with VIP scores ≥ 2.5 in inflorescences exposed to UVB, UVA, or blue light. Positive or negative values on the x-axis indicate higher and lower relative abundance compared with the dataset’s mean. Boxplots represent median (horizontal line), interquartile range (box), mean (yellow diamond), maximum and minimum (whiskers), and outliers (beyond whiskers). Mean values represent the average of six inflorescences per treatment (*n* = 6). Different letters indicate significantly different values for short-wavelength treatments according to a Fisher’s protected LSD test (α = 0.05)

After identifying enriched flavonoid motifs, we assessed the effect of short-wavelengths on the accumulation of distinct flavonoid subclasses. Notably, UVB exposure was associated with a magenta coloration in glandular trichomes on bracts (Fig. 3A), a phenotypic change absent under the other treatments (Supplementary Fig. 8A-C). PLS-DA of the phenylpropanoid/shikimate dataset generated a model in which the first two components significantly contributed to clustering based on short-wavelength treatment (Component 1: 7.8%; Component 2: 16.5%) (Fig. 3B). Model quality metrics confirmed the robust performance R^2^ = 0.98, Q^2^ = 0.52, and accuracy 0.84 (Supplementary Fig. 9 A). In Fig. 3, we show the top features with > 2.5 VIP scores with their relative abundances (Fig. 3C-D). To expand our analysis on the number of most differentially affected futures, we focused on a total of 15 features with VIP scores ≥ 1.5 contributed to separation of clusters observed in the PLS-DA (Supplementary Fig. 9B). From those metabolite features, 10 belong to the flavonoid super class, including five flavones (ID: 2523, putatively annotated as tricetin MSI-level 3; ID: 2291 MSI-level 3 putatively annotated as vicenin 2 | VIP: 3.4, 2.8 | mass: 303.049 *m/z*; 595.165 *m/z;* ID: 6168 MSI-level unresolved; 5265 MSI-level 3 unresolved), two flavonols (ID: 2662; 2617 putatively annotated as isorhamnetin-3-O-rutinoside and kaempferol-3-O-glucuronoside at a MSI-level 3 | VIP: 4.6; 3.3 | mass: 231.049 *m/z*; 463.087 *m/z*), one anthocyanidin (ID: 2627 MSI-level 3 unresolved identity; VIP: 2.9; mass: 1177.396 *m/z*), and two flavanones (ID: 5377 MSI-level 3 unresolved identity; VIP: 2.5; mass: 371.149 *m/z* and ID: 5352 MSI-level 3 unresolved identity; VIP: 1.5; mass: 469.185 *m/z*) (Fig. 3D) (for further details see Supplementary Table 1). A clustering heat map of the phenylpropanoid dataset revealed that the flavonoids subclass showed similar abundances in response to UVB and blue light (Fig. 3C), which is in accordance with previous findings on total leaf flavonoids (Kotiranta et al., 2024).

UVA significantly increased relative abundances of six flavonoids including two flavones (ID: 2291 putatively annotated as vicenin 2; 5265 MSI-level 3 unresolved identity) and two flavanones (ID: 5377 MSI-level 3 unresolved identity; 5352 MSI-level 3 unresolved identity) and decreased a flavone (ID: 2435 putatively annotated as vitexin MSI-level 2 | Log_2_FC: −1.1) and flavonol (ID: 2662, putatively annotated as isorhamnetin-3-O-rutinoside) (Table 1, Supplementary Table 1 for further details; Supplementary Fig. 10B). Finally, blue light significantly increased relative abundances of four flavonoids including flavonol (ID: 2617, putatively annotated as kaempferol-3-O-glucuronoside), flavone (ID: 2523 putatively annotated as tricetin), anthocyanidin (ID: 2627, MSI-level 3), and flavanone (ID: 5377 MSI-level 3) (Table 1; Supplementary Table 1 for further details; Supplementary Fig. 10 C). Altogether, these results suggest that among identified flavonoid classes, flavones and flavonols are the most strongly induced in response to short wavelengths. UVB and blue light upregulate similar flavonoid subclasses (flavonol, flavone, anthocyanidin), whereas UVB and UVA downregulate flavonol accumulation. In contrast, UVA and blue light promote flavanone production. Overall, the magnitude of treatment-associated changes was greatest under UVB, with blue showing partial overlap and UVA producing a distinct subclass profile.

Notably, the most strongly UVB-associated features within the phenylpropanoid/shikimate class were higher-mass nodes and flavonoid-like signals, suggesting that post-biosynthetic modification (including glycosylation) may be a major driver of the UVB response. We therefore next examined flavonoid subclasses and glycosylation patterns in detail.

Short-wavelength-dependent accumulation of glycosylated flavonoids in cannabis inflorescences.

**Fig. 4:**
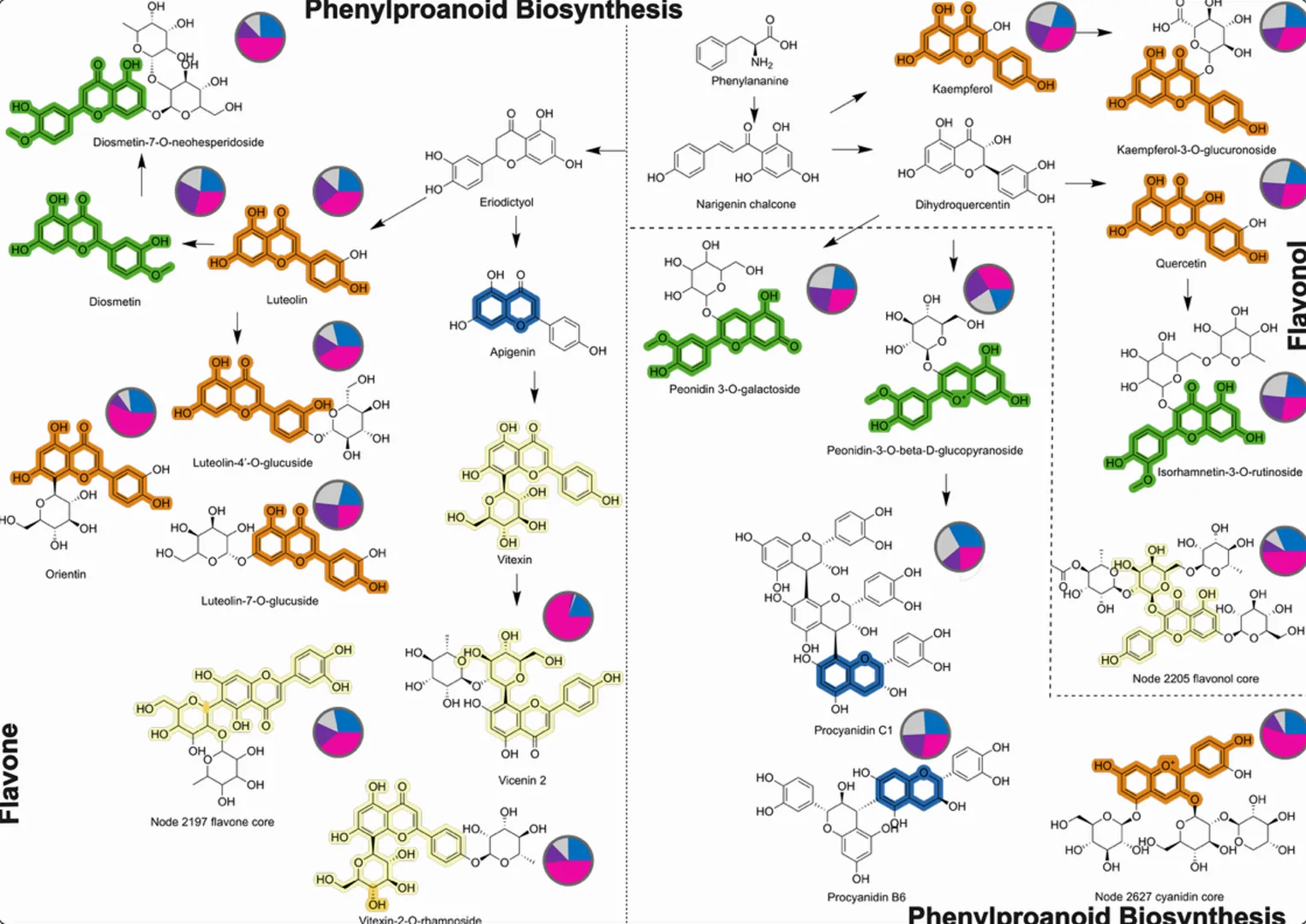
Effect of short-wavelength (UVB, UVA, blue light) on the biosynthesis of flavones, flavonols, and anthocyanins in *Cannabis sativa* L. inflorescences. A simplified overview of the phenylpropanoid biosynthesis focused in the downstream section responsible to produce flavonoids, for further details in each node see Supplementary Table 1. Chemical structures highlighted in green, orange, light-yellow and blue correspond to motif_68, motif_59, motif_137, and motif_105, respectively, representing shared substructures between flavonoid subclass (flavone, flavonol, and anthocyanin). Pie charts represent relative abundances for each identified feature (compound) according to the feature-based molecular network in positive ionization (FBMN-POS), where each colour represents UVB (pink), UVA (purple) and blue (blue) light treatments, and reference or control (grey)

To assess the effect of UVB, UVA, and blue light on the accumulation of flavonoids subclasses, we focused on identifying glycosylation patterns associated to flavanols, flavones, and anthocyanins. Several members of the flavonoid biosynthetic pathway were detected in the cannabis floral metabolome, belonging to downstream branches originating from phenylalanine (Fig. 4). Some of the early intermediates in the flavonoid pathway including the putatively compounds: apigenin (MSI-level 3, node 4146), eriodictyol (MSI-level 3, node 1205) could be identified but were not differentially affected by UV or blue light treatments. Numerous flavones and flavonols were identified, predominantly in glycosylated forms.

Comparative analysis of flavonoid relative abundances across short-wavelength treatments revealed a clear divergence between aglycones and glycosylated derivatives (Fig. 4). Putative non-glycosylated flavonoids, including luteolin, kaempferol, and apigenin, did not exhibit significant differences in relative abundances in response to UVB, UVA, and blue light treatments. In contrast, several glycosylated flavonoids, such as luteolin 7-glucoside (MSI-level 3), luteolin-4’-O-glucoside (MSI-level 3), kaempferol-3-O-glucoside (MSI-level 2), vitexin (MSI-level 2), orientin (MSI-level 3) and others (see Supplementary Table 1.). These results indicate UVB preferentially promotes flavonoid glycosylation rather than increasing abundances of the corresponding aglycone backbones.

Importantly, both O-glycosylated flavonols (e.g., luteolin-4’-O-glucoside and kaempferol-3-O-glucoside) and C-glycosylated flavones (e.g., vitexin and orientin) were enhanced under UVB. This concurrent accumulation of distinct glycosylation types suggests that UVB exposure broadly affects flavonoid glycosylation across structurally and biosynthetically distinct subclasses, rather than selectively inducing a single glycosylation route.

**Table 1:** Effect of short wavelength (UVB, UVA, blue light) on flavonoids relative abundances in inflorescences from eight weeks after the onset of flowering of *Cannabis sativa* L.

ID					Log_2_FC^§^		
	VIP Score	MSI-level	Chemical class^#^	Mass (m/z)	UVB vs. Ref	UVA vs. Ref	Blue vs. Ref
2662	4.6	3	Flavonol	625.176	−2.1^***^	−1.9^***^	–
2617	3.4	3	Flavonol	463.087	3.2^***^	–	2.0^**^
2523	3.3	3	Flavone	303.049	2.7^***^	–	1.3^**^
2627	2.9	3	Anthocyanidin	1177.396	2.3^***^	–	1.2^**^
2291	2.8	3	Flavone	595.165	–	1.6^***^	–
5377	2.5	3	Flavanone	371.149	–	1.4^***^	1.2^*^
6168	2.1	3	Flavone	283.062	1.1^*^	–	–
5265	1.9	3	Flavone	469.185	–	2.8^***^	–
2435	1.7	2	Flavone	433.112	–	−1.1^*^	–
5352	1.5	3	Flavanone	469.185	–	5.8^***^	–

§Log_2_fold-change (FC) values obtained from volcano plot analysis where the short-wavelength treatments were compared to the reference. Positive and negative values indicate upregulation and downregulation of relative abundances, respectively. VIP scores obtained from the partial least squared – discriminant analysis (PLS-DA) of the phenylpropanoid/shikimate dataset. *p-value < 0.05; **p-value < 0.01; ***p-value < 0.001. MSI-level stands for Metabolomics Standards Initiative (MSI) levels. ^#^Chemical class level is retrieved via MS2Query

Metabolic features displaying the highest relative abundances under UVB were predominantly glycosylated and frequently of high molecular mass, most notably motif_137 (annotated as glycosylated flavonoid substructure), present in the flavonoid molecular family. Among these were orientin, luteolin-4’-O-glucoside, vitexin, and kaempferol-3-O-glucoside, as well as three high-mass molecular network nodes (ID: 2205; 2197; 2627), all exceeding 1000 Da (Fig. 3). Metabolic feature 2205 (precursor *m/z* 1029.344, MSI-level 3) showed a library match to O-β-D-xylopyranosylorientin and structurally related analogues, although no exact mass match was obtained. Metabolic feature 2197 (*m/z* 1189.323, MSI-level 3) lacked direct library matches but was annotated by MS2Query analogue search as a highly oxygenated flavone with multiple sugar moieties (“Flavone base + 4O, C-Hex-dHex”), and contained motif_137, supporting its classification as a glycosylated flavonoid. Metabolic feature 2627 (*m/z* 1177.396, MSI-level 3) showed a putative annotation as a highly glycosylated anthocyanidin structure and contained motif_59, reinforcing its placement as an anthocyanidin derivate within the broader flavonoid family. Although precise structural elucidation of these high-mass compounds was not achieved, their shared substructural features and strong responsiveness to UVB exposure indicate a coordinated accumulation of extensively glycosylated flavonoids.

Collectively, the combined abundance patterns and substructure-level evidence demonstrate that UVB selectively enhances both the abundance and structural complexity of glycosylated flavonoids in cannabis inflorescences. The enrichment of both C- and O-glycosylated flavonoids under UVB supports the hypothesis that UVB-driven modulation of the floral metabolome operates primarily through post-biosynthetic diversification of flavonoids, potentially expanding the antioxidant pool available for cellular compartmentalization and redox homeostasis under UV-induced stress.

## Discussion

Here, we demonstrated how a comparative computational metabolomic workflow characterized the cannabis floral metabolome by library annotations based on analogues, chemical classes predictions, and sub-structural scaffolds assignments. Based on VIP scores ≥ 1.5, short wavelength treatments affected at least seven major biochemical classes including polyketides, amino acids and peptides, terpenoids, fatty acids, alkaloids, carbohydrates, and phenylpropanoid/shikimates (see Supplementary Information 3.1). Among these, the phenylpropanoid/shikimate pathway, and more specifically the flavonoid subclass, emerged as the primary short-wavelength-responsive metabolic domain, consistent with findings in cannabis leaves (Kotiranta et al., 2024), *Brassica napus *(Lee et al., 2022), and *Oryza sativa* (Zhang et al., 2024). This selective responsiveness underscores the role of flavonoids as key mediators of plant acclimation to short-wavelength-associated stressors, rather than a broad metabolic response.

A key finding of this work enabled by implementing feature-based molecular networking and MS2LDA substructure discovery, is that UVB selectively increases the abundance of glycosylated flavonoids, while the corresponding aglycone backbones remain largely unchanged. Glycosylation of flavonoids can occur via an oxygen or carbon bond, namely O- and C-glycosylation, respectively, and these post-biosynthetic modifications are key for flavonoids accumulation and auto-toxicity prevention (Behr et al., 2020). Both O-glycosylated flavonols and C-glycosylated flavones were enhanced under UVB treatment. Flavonol glycosides (e.g. quercetin-3-O-rutinoside) are stronger antioxidants than flavone glycosides (e.g. luteolin-7-O-glucoside), therefore plants prioritize the biosynthesis of flavonols over flavones as quenchers of potential UVB damage (Tattini et al., 2004). Moreover, O-glycosylation is typically associated with vacuolar accumulation of flavonols and fast de-glycosylation, whereas C-glycosylated flavones are more chemically stable and resistant to hydrolysis, suggesting complementary protective functions (Behr et al., 2020; Supplementary Information 3.2). The simultaneous accumulation of these structurally and biosynthetically distinct glycosylation types indicates that UVB does not selectively activate a single route, but rather promotes broad flavonoid glycosylation, involving distinct enzyme families with different substrate specificities and cellular localizations as part of a major UV-regulated and light-regulated network of the phenylpropanoid pathway (Le Roy et al., 2016).

The enrichment of glycosylated flavonoids under UVB exposure may reflect a scenario in which cannabis inflorescences increase their capacity to buffer ROS through compartmentalized glycosylated flavonoid pools, which upon oxidative cues are de-glycosylated to rapidly release aglycones capable of quenching ROS, thereby mitigating photon-induced cellular damage. The coordinated increase of flavonols and flavone flavonoid subclasses suggests upstream regulation at the transcriptional level of multiple glycosyltransferase pathways, likely via the interaction between UVR8, HY5 and MYB transcription factors, whose binding elements have been localized in glycosyltransferase promoters in rice (Clayton et al., 2018; Zhang et al., 2020). The presence of magenta pigmentation in cannabis glandular trichomes under UVB treatment further supports the notion that flavonoid accumulation contributes to localized photoprotection in reproductive tissues. A yet unannotated anthocyanin-like feature (ID: 2627) (Figs. 3D and 4) with a putative glycosylation, was strongly enhanced by UVB, making it a putative candidate responsible for this pigmentation, distinct from the cyanidin-3-rutinoside previously attributed to purple hues in cannabis (Bassolino et al., 2023).

The accumulation of such highly modified flavonoids suggests that UVB exposure not only increases flavonoid abundance but also expands structural complexity, likely enhancing functional diversity and stress robustness. UVB can therefore be used to steer the production of flavonoids in plants, impacting their medicinal and nutritional quality (Contreras-Avilés et al., 2024), as reported in *Fagopyrum esculentum* sprouts (Tian et al., 2024), *Ocimum basilicum* (Skowron et al., 2024), micro-tomato leaves (Lima et al., 2023), and others (Li et al., 2021; Zhu et al., 2023).

Interestingly, the lack of detectable changes in cannabinoid abundances across short-wavelength treatments contrasts with earlier measurements showing that UV radiation broadly enhances cannabis specialized metabolism (Jenkins, 2021; Li et al., 2025; Lydon et al., 1987), reinforcing the idea that cannabinoids and flavonoids fulfil distinct physiological roles in UV-stress adaptation (Agati & Tattini, 2010; Li et al., 2025). Based on our analyses, we could not detect prenylated flavones (cannflavins), nor changes in cannabinoids abundances in the chemotype II (balanced THC: CBD) cannabis genotype used, suggesting a genotype-specific response. Cannflavin abundances are mainly determined by genetics and low air temperatures (6–16 °C) at 1200 m above sea level (Calzolari et al., 2017; Giupponi et al., 2020). Accumulation of cannflavins has been reported in chemotypes I (high THC) (Radwan et al., 2008), as well in chemotypes III (low THC) (Cerrato et al., 2023; Giupponi et al., 2020; Kotiranta et al., 2024). With regards to cannabinoids abundances, substantial responses of cannabinoids levels to UVB were only reported in cannabis THC-dominant genotypes (chemotype I) (Li et al., 2025; Lydon et al., 1987) supporting the idea that the genetic makeup should be considered as a major determinant of cannabis responses to short wavelengths. Nevertheless, our findings are insufficient to conclude that a chemotype II does not produce cannflavins or that cannabinoids abundances are irresponsive to short wavelengths. Other factors including higher short wavelength doses at different development timings (earlier in the flowering phase) should not be overlooked.

Beyond physiological insights, our study highlights how integrated computational metabolomics can connect environmental triggers (e.g., light) to pathway-level classes and post-biosynthetic enzymatic modifications. The combination of FBMN, MS2LDA, SIRIUS/CANOPUS, and deep learning similarity methods (MS2Query and DreaMS) provided the multi-layer evidence needed to interpret treatment effects at the level of flavonoid subclasses and glycosylation types, where exact library matches alone would have resulted in incomplete annotations (van der Hooft et al., 2011). Structural assignments should nonetheless be regarded as putative, as flavonoid isomers can remain indistinguishable by MS/MS alone, and mechanistic claims regarding compartmentalization and regulation will benefit from integration with transcriptomic data and targeted MS/MS of diagnostic glycosides. Although the untargeted nature of this study did not permit direct assessment of bioactivity, glycosylated flavonoids, particularly flavone-C glycosides, have been explicitly linked to high tissue antioxidant capacity in *Cannabis sativa* (Chen et al., 2024). Ultimately, determining whether the UV-induced accumulation of these specific compounds translates to measurable improvements in the localized antioxidant activity of the floral tissue represents exciting venues for future research. Elucidating these relationships will not only strengthen our understanding of cannabis physiology but also optimize environmental cultivation strategies aimed at maximizing phytochemical quality.

## Conclusion

In conclusion, our results demonstrate that UVB radiation selectively reshapes the cannabis floral metabolome by promoting the accumulation and structural diversification of flavonoids without any apparent effect on cannabinoids, while UVA does not substantially affect the cannabis floral flavonoid metabolome. The concurrent induction of C- and O-glycosylated flavonoids, supported by substructure-level metabolomic evidence, indicates that UVB-driven acclimation involves coordinated regulation of flavonoid post-biosynthetic modification pathways, advancing our understanding of UV- and light-mediated metabolic plasticity in cannabis. The integrative metabolomics framework developed here can be applied to characterize plant metabolic responses to environmental conditions beyond light, across other plant tissues and natural product classes. Finally, our findings suggest that UVB supplementation in controlled-environment cannabis cultivation may be used to enhance glycosylated flavonoid accumulation, potentially impacting inflorescence medicinal value independently of cannabinoid content, opening new avenues for fine-tuning light spectra in controlled environments to optimize for the presence of specific chemicals in medicinal cannabis.

## Supplementary Information

Below is the link to the electronic supplementary material.

Supplementary Material 1

## Data Availability

The authors confirm that the data supporting the findings of this study are available within the article, supporting information, or dedicated repositories. All raw and processed metabolomics data are publically available in the MassIVE ID: MSV000100919 including the pre-processed files, metadata and quantification tables. The batch file (mzmine), final annotation table for the flavonoid molecules, GNPS networks (graphML files, job IDs: positive 11072d72767f4e73923333bb0661d5c9 and negative 9a3d6eaf1463432c9fb00c31830031ef), SIRIUS, MS2Query, DreaMS and MS2LDA results, and script used are available at Zenodo (ID: 18715236, https://zenodo.org/records/18715236).
